# 2-(5,7-Dimethyl-3-methyl­sulfanyl-1-benzofuran-2-yl)acetic acid

**DOI:** 10.1107/S1600536808022988

**Published:** 2008-07-26

**Authors:** Hong Dae Choi, Pil Ja Seo, Byeng Wha Son, Uk Lee

**Affiliations:** a Department of Chemistry, Dongeui University, San 24 Kaya-dong, Busanjin-gu, Busan 614-714, Republic of Korea; b Department of Chemistry, Pukyong National University, 599-1 Daeyeon 3-dong, Nam-gu, Busan 608-737, Republic of Korea

## Abstract

The title compound, C_13_H_14_O_3_S, was prepared by alkaline hydrolysis of ethyl 2-(5,7-dimethyl-3-methyl­sulfanyl-1-benzofuran-2-yl)acetate. In the crystal structure, the carboxyl groups are involved in inter­molecular O—H⋯O hydrogen bonds, which link the mol­ecules into centrosymmetric dimers. These dimers are further packed into stacks along the *a* axis by weak C—H⋯π inter­actions.

## Related literature

For the crystal structures of similar 2-(3-methyl­sulfanyl-1-benzofuran-2-yl)acetic acid derivatives, see: Choi *et al.* (2007 ▶); Seo *et al.* (2007 ▶).
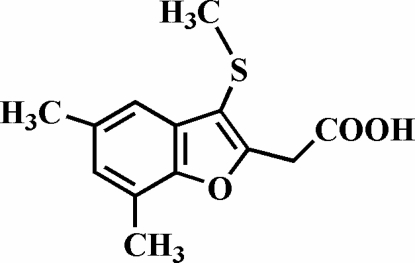


## Experimental

### 

#### Crystal data

C_13_H_14_O_3_S
*M* *_r_* = 250.30
Triclinic, 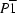

*a* = 4.7225 (9) Å
*b* = 7.476 (2) Å
*c* = 17.687 (3) Å
α = 80.91 (3)°
β = 89.86 (3)°
γ = 80.67 (3)°
*V* = 608.3 (2) Å^3^
*Z* = 2
Mo *K*α radiation
μ = 0.26 mm^−1^
*T* = 293 (2) K
0.40 × 0.40 × 0.20 mm

#### Data collection

Bruker SMART CCD diffractometer
Absorption correction: none
4707 measured reflections
2344 independent reflections
2156 reflections with *I* > 2σ(*I*)
*R*_int_ = 0.059

#### Refinement

*R*[*F* ^2^ > 2σ(*F* ^2^)] = 0.047
*wR*(*F* ^2^) = 0.144
*S* = 1.24
2344 reflections
161 parameters
H atoms treated by a mixture of independent and constrained refinement
Δρ_max_ = 0.38 e Å^−3^
Δρ_min_ = −0.41 e Å^−3^

### 

Data collection: *SMART* (Bruker, 2001 ▶); cell refinement: *SAINT* (Bruker, 2001 ▶); data reduction: *SAINT*; program(s) used to solve structure: *SHELXS97* (Sheldrick, 2008 ▶); program(s) used to refine structure: *SHELXL97* (Sheldrick, 2008 ▶); molecular graphics: *ORTEP-3* (Farrugia, 1997 ▶) and *DIAMOND* (Brandenburg, 1998 ▶); software used to prepare material for publication: *SHELXL97*.

## Supplementary Material

Crystal structure: contains datablocks global, I. DOI: 10.1107/S1600536808022988/cv2433sup1.cif

Structure factors: contains datablocks I. DOI: 10.1107/S1600536808022988/cv2433Isup2.hkl

Additional supplementary materials:  crystallographic information; 3D view; checkCIF report

## Figures and Tables

**Table 1:** Hydrogen-bond geometry (Å, °) *Cg* is the centroid of the C2–C7 ring.

*D*—H⋯*A*	*D*—H	H⋯*A*	*D*⋯*A*	*D*—H⋯*A*
O3—H3O⋯O2^i^	0.73 (4)	1.95 (4)	2.680 (3)	177 (4)
C9—H9*A*⋯*Cg*^ii^	0.96	2.72	3.621 (4)	156

Symmetry codes: (i) 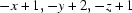; (ii) 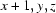.

**Fig. 1.**
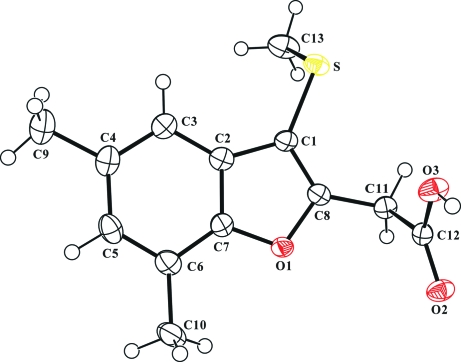
The molecular structure of the title compound, showing displacement ellipsoids drawn at the 50% probability level.

**Fig. 2.**
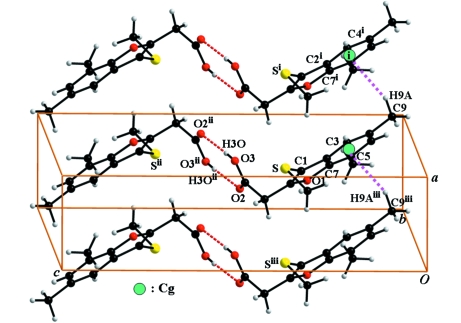
A portion of the crystal packing showing C—H···π interaction and hydrogen bonds (dotted lines). Cg denotes the C2–C7 ring centroid [symmetry codes: (i) x + 1, y, z; (ii) -x + 1, -y + 2, -z + 1; (iii) x - 1, y, z].

## supplementary crystallographic information

### Comment

As a part of our ongoing studies on the synthesis and structures of 2-(3-methylsulfanyl-1-benzofuran-2-yl)acetic acid derivatives, we have described 2-(5-ethyl-3-methylsulfanyl-1-benzofuran-2-yl)acetic acid (Seo *et al.*, 2007) and 2-(3-methylsulfanyl-5-phenyl-1-benzofuran-2-yl)acetic acid (Choi *et al.*, 2007). Here we report the crystal structure of the title compound, (I) (Fig. 1).

In (I), the benzofuran unit is essentially planar, with a mean deviation of 0.004 (2) Å from the least-squares plane defined by the nine constituent atoms. The crystal packing (Fig. 2) is stabilized by classical inversion-related O—H···O hydrogen bonds (Table 1) and C—H···π interactions between a methyl H atom and the ring C2–C7 (Cg is its centroid) of a neighbouring molecule (Table 1).

### Experimental

Ethyl 2-(5,7-dimethyl-3-methylsulfanyl-1-benzofuran-2-yl)acetate (417 mg, 1.50 mmol) was added to a solution of potassium hydroxide (505 mg, 9.0 mmol) in water (25 ml) and methanol (25 ml), and the mixture was refluxed for 5 h, then cooled. Water was added, and the solution was extracted with dichloromethane. The aqueous layer was acidified to pH 1 with concentrated hydrochloric acid and then extracted with chloroform, dried over magnesium sulfate, filtered and concentrated under vacuum. The residue was purified by column chromatography (ethyl acetate) to afford the title compound as a colorless solid [yield 82%, m.p. 420–421 K; *R*_f_ = 0.66 (ethyl acetate)]. Single crystals suitable for X-ray diffraction were prepared by evaporation of a solution of the title compound in benzene at room temperature. Spectroscopic analysis: ^1^H NMR (CDCl_3_, 400 MHz) δ 2.32 (s, 3H), 2.43 (s, 3H), 2.45 (s, 3H), 4.03 (s, 2H), 6.93 (s, 1H), 7.25 (s, 1H), 10.10 (s, 1H); EI—MS 250 [*M*^+^].

### Refinement

Atom H3O of the hydroxy group was found in a difference Fourier map and refined isotropically. The other H atoms were positioned geometrically and refined using a riding model, with C—H = 0.95 Å for aromatic H atoms, 0.97 Å for methylene H atoms and 0.96 Å for methyl H atoms, respectively, and with *U*_iso_(H) = 1.2Ueq(C) for aromatic and methylene H atoms and 1.5Ueq(C) for methyl H atoms.

### Crystal data

**Table:**

C_13_H_14_O_3_S	*Z* = 2
*M**_r_* = 250.30	*F*_000_ = 264
Triclinic, *P*1	*D*_x_ = 1.367 Mg m^−^^3^
Hall symbol: -P_1	Melting point = 420–421 K
*a* = 4.7225 (9) Å	Mo *K*α radiation λ = 0.71073 Å
*b* = 7.476 (2) Å	Cell parameters from 3835 reflections
*c* = 17.687 (3) Å	θ = 2.3–28.3º
α = 80.91 (3)º	µ = 0.26 mm^−^^1^
β = 89.86 (3)º	*T* = 293 (2) K
γ = 80.67 (3)º	Block, colourless
*V* = 608.3 (2) Å^3^	0.40 × 0.40 × 0.20 mm

### Data collection

**Table:**

Bruker SMART CCD diffractometer	2344 independent reflections
Radiation source: fine-focus sealed tube	2156 reflections with *I* > 2σ(*I*)
Monochromator: graphite	*R*_int_ = 0.059
Detector resolution: 10.0 pixels mm^-1^	θ_max_ = 26.0º
*T* = 293(2) K	θ_min_ = 1.2º
φ and ω scans	*h* = −5→5
Absorption correction: none	*k* = −9→9
4707 measured reflections	*l* = −21→21

### Refinement

**Table:**

Refinement on *F*^2^	Secondary atom site location: difference Fourier map
Least-squares matrix: full	Hydrogen site location: inferred from neighbouring sites
*R*[*F*^2^ > 2σ(*F*^2^)] = 0.047	H atoms treated by a mixture of independent and constrained refinement
*wR*(*F*^2^) = 0.144	*w* = 1/[σ^2^(*F*_o_^2^) + (0.063*P*)^2^ + 0.3562*P*] where *P* = (*F*_o_^2^ + 2*F*_c_^2^)/3
*S* = 1.24	(Δ/σ)_max_ < 0.001
2344 reflections	Δρ_max_ = 0.38 e Å^−^^3^
161 parameters	Δρ_min_ = −0.41 e Å^−^^3^
Primary atom site location: structure-invariant direct methods	Extinction correction: none

### Special details

**Table:**

Geometry. All e.s.d.'s (except the e.s.d. in the dihedral angle between two l.s. planes) are estimated using the full covariance matrix. The cell e.s.d.'s are taken into account individually in the estimation of e.s.d.'s in distances, angles and torsion angles; correlations between e.s.d.'s in cell parameters are only used when they are defined by crystal symmetry. An approximate (isotropic) treatment of cell e.s.d.'s is used for estimating e.s.d.'s involving l.s. planes.
Refinement. Refinement of *F*^2^ against ALL reflections. The weighted *R*-factor *wR* and goodness of fit *S* are based on *F*^2^, conventional *R*-factors *R* are based on *F*, with *F* set to zero for negative *F*^2^. The threshold expression of *F*^2^ > σ(*F*^2^) is used only for calculating *R*-factors(gt) *etc*. and is not relevant to the choice of reflections for refinement. *R*-factors based on *F*^2^ are statistically about twice as large as those based on *F*, and *R*- factors based on ALL data will be even larger.

### Fractional atomic coordinates and isotropic or equivalent isotropic displacement parameters (Å^2^)

**Table:**

	*x*	*y*	*z*	*U*_iso_*/*U*_eq_	
S	0.85977 (13)	0.34264 (8)	0.36615 (3)	0.0254 (2)	
O1	0.4166 (3)	0.8236 (2)	0.27417 (9)	0.0223 (4)	
O2	0.2357 (4)	0.9616 (2)	0.44893 (10)	0.0272 (4)	
O3	0.6544 (4)	0.7728 (2)	0.47532 (11)	0.0282 (4)	
H3O	0.685 (8)	0.848 (5)	0.494 (2)	0.049 (11)*	
C1	0.6982 (5)	0.5473 (3)	0.30908 (14)	0.0214 (5)	
C2	0.7803 (5)	0.6242 (3)	0.23378 (14)	0.0232 (5)	
C3	0.9867 (5)	0.5690 (3)	0.18203 (15)	0.0268 (5)	
H3	1.1098	0.4574	0.1936	0.032*	
C4	1.0055 (6)	0.6834 (4)	0.11284 (15)	0.0293 (6)	
C5	0.8179 (6)	0.8526 (3)	0.09713 (15)	0.0306 (6)	
H5	0.8332	0.9275	0.0505	0.037*	
C6	0.6115 (6)	0.9133 (3)	0.14758 (15)	0.0279 (5)	
C7	0.6010 (5)	0.7931 (3)	0.21521 (14)	0.0231 (5)	
C8	0.4821 (5)	0.6706 (3)	0.32972 (13)	0.0211 (5)	
C9	1.2248 (6)	0.6283 (4)	0.05502 (17)	0.0400 (7)	
H9A	1.4113	0.6436	0.0718	0.060*	
H9B	1.1762	0.7043	0.0063	0.060*	
H9C	1.2265	0.5021	0.0502	0.060*	
C10	0.4134 (6)	1.0944 (3)	0.13076 (17)	0.0359 (6)	
H10A	0.2325	1.0833	0.1544	0.054*	
H10B	0.3842	1.1291	0.0764	0.054*	
H10C	0.4972	1.1864	0.1508	0.054*	
C11	0.3130 (5)	0.6760 (3)	0.40038 (13)	0.0225 (5)	
H11A	0.3482	0.5562	0.4324	0.027*	
H11B	0.1099	0.7050	0.3869	0.027*	
C12	0.3944 (5)	0.8187 (3)	0.44444 (13)	0.0195 (5)	
C13	0.7336 (6)	0.1752 (3)	0.31692 (18)	0.0360 (6)	
H13A	0.5277	0.1920	0.3176	0.054*	
H13B	0.8110	0.0538	0.3421	0.054*	
H13C	0.7950	0.1908	0.2649	0.054*	

### Atomic displacement parameters (Å^2^)

**Table:**

	*U*^11^	*U*^22^	*U*^33^	*U*^12^	*U*^13^	*U*^23^
S	0.0300 (3)	0.0185 (3)	0.0271 (3)	−0.0028 (2)	−0.0065 (2)	−0.0025 (2)
O1	0.0275 (8)	0.0163 (7)	0.0230 (9)	−0.0025 (6)	0.0001 (6)	−0.0039 (6)
O2	0.0275 (9)	0.0214 (8)	0.0334 (10)	−0.0003 (7)	−0.0053 (7)	−0.0105 (7)
O3	0.0251 (9)	0.0248 (9)	0.0370 (10)	−0.0017 (7)	−0.0069 (7)	−0.0140 (8)
C1	0.0254 (11)	0.0162 (10)	0.0241 (12)	−0.0044 (8)	−0.0027 (9)	−0.0063 (9)
C2	0.0256 (12)	0.0200 (11)	0.0263 (12)	−0.0079 (9)	−0.0025 (9)	−0.0066 (9)
C3	0.0267 (12)	0.0261 (12)	0.0297 (13)	−0.0068 (9)	0.0006 (10)	−0.0084 (10)
C4	0.0334 (13)	0.0314 (13)	0.0289 (13)	−0.0150 (10)	0.0057 (10)	−0.0125 (10)
C5	0.0418 (15)	0.0292 (13)	0.0235 (13)	−0.0155 (11)	0.0006 (10)	−0.0024 (10)
C6	0.0366 (13)	0.0209 (11)	0.0281 (13)	−0.0092 (10)	−0.0021 (10)	−0.0051 (10)
C7	0.0280 (12)	0.0209 (11)	0.0227 (12)	−0.0074 (9)	−0.0006 (9)	−0.0066 (9)
C8	0.0256 (11)	0.0170 (10)	0.0223 (11)	−0.0066 (8)	−0.0045 (9)	−0.0045 (9)
C9	0.0449 (16)	0.0437 (16)	0.0368 (15)	−0.0173 (13)	0.0140 (13)	−0.0125 (13)
C10	0.0480 (16)	0.0247 (13)	0.0318 (14)	−0.0035 (11)	−0.0049 (12)	0.0031 (11)
C11	0.0252 (11)	0.0191 (10)	0.0253 (12)	−0.0074 (9)	0.0000 (9)	−0.0059 (9)
C12	0.0218 (11)	0.0188 (10)	0.0193 (11)	−0.0071 (8)	0.0025 (8)	−0.0035 (8)
C13	0.0439 (15)	0.0200 (11)	0.0452 (16)	−0.0068 (11)	−0.0132 (12)	−0.0066 (11)

### Geometric parameters (Å, °)

**Table:**

S—C1	1.755 (2)	C5—H5	0.9300
S—C13	1.808 (3)	C6—C7	1.384 (4)
O1—C8	1.379 (3)	C6—C10	1.503 (4)
O1—C7	1.380 (3)	C8—C11	1.484 (3)
O2—C12	1.215 (3)	C9—H9A	0.9600
O3—C12	1.316 (3)	C9—H9B	0.9600
O3—H3O	0.73 (4)	C9—H9C	0.9600
C1—C8	1.351 (3)	C10—H10A	0.9600
C1—C2	1.443 (3)	C10—H10B	0.9600
C2—C7	1.393 (3)	C10—H10C	0.9600
C2—C3	1.394 (3)	C11—C12	1.514 (3)
C3—C4	1.389 (4)	C11—H11A	0.9700
C3—H3	0.9300	C11—H11B	0.9700
C4—C5	1.410 (4)	C13—H13A	0.9600
C4—C9	1.509 (4)	C13—H13B	0.9600
C5—C6	1.390 (4)	C13—H13C	0.9600
			
C1—S—C13	100.58 (12)	C4—C9—H9A	109.5
C8—O1—C7	105.62 (18)	C4—C9—H9B	109.5
C12—O3—H3O	109 (3)	H9A—C9—H9B	109.5
C8—C1—C2	106.3 (2)	C4—C9—H9C	109.5
C8—C1—S	126.10 (19)	H9A—C9—H9C	109.5
C2—C1—S	127.50 (18)	H9B—C9—H9C	109.5
C7—C2—C3	119.3 (2)	C6—C10—H10A	109.5
C7—C2—C1	105.7 (2)	C6—C10—H10B	109.5
C3—C2—C1	135.1 (2)	H10A—C10—H10B	109.5
C4—C3—C2	118.9 (2)	C6—C10—H10C	109.5
C4—C3—H3	120.6	H10A—C10—H10C	109.5
C2—C3—H3	120.6	H10B—C10—H10C	109.5
C3—C4—C5	119.3 (2)	C8—C11—C12	110.56 (18)
C3—C4—C9	120.6 (3)	C8—C11—H11A	109.5
C5—C4—C9	120.1 (2)	C12—C11—H11A	109.5
C6—C5—C4	123.5 (2)	C8—C11—H11B	109.5
C6—C5—H5	118.3	C12—C11—H11B	109.5
C4—C5—H5	118.3	H11A—C11—H11B	108.1
C7—C6—C5	114.6 (2)	O2—C12—O3	124.3 (2)
C7—C6—C10	122.1 (2)	O2—C12—C11	122.7 (2)
C5—C6—C10	123.3 (2)	O3—C12—C11	112.94 (19)
O1—C7—C6	125.3 (2)	S—C13—H13A	109.5
O1—C7—C2	110.3 (2)	S—C13—H13B	109.5
C6—C7—C2	124.4 (2)	H13A—C13—H13B	109.5
C1—C8—O1	112.1 (2)	S—C13—H13C	109.5
C1—C8—C11	132.8 (2)	H13A—C13—H13C	109.5
O1—C8—C11	115.00 (19)	H13B—C13—H13C	109.5
			
C13—S—C1—C8	110.5 (2)	C10—C6—C7—O1	−0.8 (4)
C13—S—C1—C2	−74.3 (2)	C5—C6—C7—C2	0.5 (3)
C8—C1—C2—C7	0.2 (2)	C10—C6—C7—C2	−179.7 (2)
S—C1—C2—C7	−175.77 (17)	C3—C2—C7—O1	−178.87 (19)
C8—C1—C2—C3	179.0 (2)	C1—C2—C7—O1	0.2 (2)
S—C1—C2—C3	3.0 (4)	C3—C2—C7—C6	0.1 (3)
C7—C2—C3—C4	−0.7 (3)	C1—C2—C7—C6	179.1 (2)
C1—C2—C3—C4	−179.4 (2)	C2—C1—C8—O1	−0.5 (2)
C2—C3—C4—C5	0.6 (3)	S—C1—C8—O1	175.57 (15)
C2—C3—C4—C9	−179.7 (2)	C2—C1—C8—C11	−177.8 (2)
C3—C4—C5—C6	0.0 (4)	S—C1—C8—C11	−1.8 (4)
C9—C4—C5—C6	−179.6 (2)	C7—O1—C8—C1	0.5 (2)
C4—C5—C6—C7	−0.6 (4)	C7—O1—C8—C11	178.39 (17)
C4—C5—C6—C10	179.6 (2)	C1—C8—C11—C12	108.4 (3)
C8—O1—C7—C6	−179.4 (2)	O1—C8—C11—C12	−68.9 (2)
C8—O1—C7—C2	−0.4 (2)	C8—C11—C12—O2	109.1 (2)
C5—C6—C7—O1	179.4 (2)	C8—C11—C12—O3	−69.6 (2)

### Hydrogen-bond geometry (Å, °)

**Table:**

*D*—H···*A*	*D*—H	H···*A*	*D*···*A*	*D*—H···*A*
O3—H3O···O2^i^	0.73 (4)	1.95 (4)	2.680 (3)	177 (4)
C9—H9A···Cg^ii^	0.96	2.72	3.621 (4)	156

Symmetry codes: (i) −*x*+1, −*y*+2, −*z*+1; (ii) *x*+1, *y*, *z*.
